# Research staff’s experiences of how the COVID-19 pandemic impacted recruitment for a paediatric network study

**DOI:** 10.1080/17482631.2024.2419158

**Published:** 2024-10-22

**Authors:** Isobel Fishman, Suzanne Henderson, Christina Vadeboncoeur

**Affiliations:** a Faculty of Medicine, University of Ottawa Faculty of Medicine, Ottawa, ON, Canada; b Department of Pediatrics, Children’s Hospital of Eastern Ontario Research Institute, Ottawa, ON, Canada; c Department of Pediatrics, University of Ottawa, Ottawa, ON, Canada

**Keywords:** Study recruitment, COVID-19 pandemic, research, research challenges, pediatric, healthcare

## Abstract

**Purpose:**

Since the COVID-19 pandemic, a paediatric network study with clinical sites across Canada suffered a reduction in participation. When research studies fail to meet enrolment targets, it can reduce the strength and validity of the results. This study explores research staff’s experiences of how the COVID-19 pandemic impacted recruitment for a paediatric network study.

**Methods:**

This study was conducted using a qualitative design. Focus group sessions were used to gain the perspective of research staff involved in recruitment and transcripts were analysed using Colaizzi’s seven-step method of data analysis.

**Results:**

Analysis revealed four major themes: (1) the COVID-19 pandemic had an impact on research activity; (2) families of children with medical complexity perform a risk-benefit assessment when deciding whether to take part in research; (3) a trusting relationship with clinicians is a key factor in research recruitment; and (4) research needs to be flexible in order to adapt to evolving contexts.

**Conclusion:**

This study identified both COVID-19 and non-COVID-19-related factors that impacted study recruitment for a paediatric network study. Understanding and addressing these challenges will mitigate the negative impacts on health outcomes that can occur when research studies fail to meet enrolment targets.

## Introduction

The COVID-19 outbreak was declared a pandemic by the World Health Organization on 11 March 2020 (World Health Organization). Since first being detected in Canada, COVID-19 has caused wide-scale disruption in the lives of citizens around the country. Provincial governments began enacting strict public health guidelines in an attempt to slow the spread of the virus, including stay-at-home orders, school and daycare closures, and non-essential business closures (Closure of places of non-essential business, 2020). These constantly changing guidelines had an impact on research activities. Research programmes within hospitals began pausing certain activities, such as in-person recruitment efforts and visits with study participants (Limiting Research at CHEO, 2020; Rubio-San-Simón et al., 2020).

When the pandemic began, there was a reduction in study participation across the globe (Mirza et al., 2022; Rubio-San-Simón et al., 2021). This decrease in research participation was felt by a paediatric network study with four clinical sites across Canada. This network study, the Pain and Irritability of Unknown Origin (PIUO) study, examined ways to standardize investigation of children with severe neurological impairment experiencing unexplained pain and/or irritability, and required participants to have an in-person medical assessment. Since the start of the pandemic, there was a 26.5% reduction in the PIUO study recruitment rate across all four sites, despite public health measures easing in each province.

At the start of the pandemic, the health impacts of the COVID-19 virus were unknown, leading to widespread fear and anxiety for many people (Chafouleas & Iovino, 2021; Mirza et al., 2022). Parents and caregivers of children with medical complexity were especially impacted as they already experience heightened stress and anxiety levels related to their child’s health (Kowanda et al., 2021; Patton et al., 2018). As the transmissibility of the virus increased, outpatient clinics and community-based services were put on temporary hold, forcing these parents to take on additional responsibilities for their children’s health (Chafouleas & Iovino, 2021; Stiles-Shields et al., 2021; von Schulz et al., 2022). This increased burden on caregivers of children requiring specialized care has led to greater parental exhaustion as they experienced a lack of support and reduced access to healthcare services (Kowanda et al., 2021; Patton et al., 2018; Stiles-Shields et al., 2021). These families are then less likely to participate in research studies and clinical trials (Fink et al., 2020; Tripp et al., 2022).

When research studies fail to meet enrolment targets, it can reduce the strength and validity of the results (Patel et al., 2003; Sun et al., 2017). Furthermore, research pauses can have negative impacts on patient care (Unger & Xiao, 2021; Whitlock et al., 2020). Researchers need to be able to adapt their recruitment efforts to reduce the potential for negative health outcomes for patients. To do this, recruitment challenges must first be identified. The purpose of this qualitative study is to explore research staff’s experiences of how the COVID-19 pandemic impacted recruitment for a paediatric network study.

## Methods

### Design

The study employed a qualitative design using focus group discussions. This approach is congruent with the aim of this study, which is to gain the perspectives of research staff who experienced recruitment challenges since the start of the COVID-19 pandemic.

### Focus group attendees

Purposeful sampling was used on past and present research staff working on the PIUO study. Research staff were chosen as focus group attendees because they experienced recruitment challenges firsthand and were in direct contact with family members of potential participants. This sampling strategy ensured maximum variation in the perspectives and experiences held across different study sites and roles (Polit & Beck, 2017). The inclusion criteria were research staff from the network study that actively participated in recruitment efforts and had spoken to at least one potential participant directly. Research staff that did not actively participate in recruitment efforts or did not speak with at least one potential participant were excluded. Potential focus group attendees were identified by Site Leads from each site (Vancouver, Calgary, Toronto, and Ottawa) and were invited to partake in the study through email invitation by one of the research staff (I.F.). To minimize the risk of potential focus group attendees feeling obligated to participate in the study, attendees were contacted by a research staff (I.F.) who is not a part of the network study and therefore does not have a previous professional relationship with the potential attendees.

### Ethical considerations

Ethics approval was granted by the Children’s Hospital of Eastern Ontario Research Ethics Board (22/51X). Each focus group attendee electronically signed a consent form and was assured that they could withdraw their consent at any time, with no risk to their current employment.

### Data collection

Focus group sessions were used to gain the perspective and experience of research staff involved in recruitment. Focus group sessions were chosen as they are time effective, flexible, and allow attendees to freely express their experiences and perceptions of the topic (Byers & Wilcox, 1973). Furthermore, one of the advantages of focus groups is the dynamic interaction between attendees who can react to what is being said by others in the session (Polit & Beck, 2017).

Two focus group sessions were conducted through Zoom, a virtual meeting platform chosen for its cost-effectiveness, ease of use, and ability to gather attendees living in different provinces (Archibald et al., 2019). Moreover, conducting qualitative interviews through virtual platforms has been found to increase rapport between the interviewer and focus group attendees (Archibald et al., 2019). Both focus groups were moderated by the same research staff (I.F.) to ensure consistency in data collection. In an attempt to mitigate the risk of social desirability bias, the focus group moderator had no previous professional relationship with the attendees.

Two focus groups were conducted in English, each lasting approximately 1–1 ½ h. Each participant virtually attended one focus group session, which began with an introduction to the research purpose, obtaining approval for session recording, and ensuring the completion of consent forms. The moderator (I.F.) used an interview guide (Table I) to lead the focus group sessions, which included probing questions to describe focus group attendees’ experiences with recruitment before and during the COVID-19 pandemic. The discussions started by asking what study recruitment looked like before and after the pandemic was declared in March 2020. The conversation continued with how the pandemic affected research activities at each site and what recruitment looked like after restrictions were lifted. The sessions ended with a discussion on reasons potential participants did not want to participate in the research study that were shared with the research staff during recruitment.

**Table I.** Focus group interview questions.

1. How long have you worked on the study for?
2. Do you currently work on the study? If not, when did you stop working on the study?
3. What was your recruitment like before March 2020?
4. What was your recruitment like after March 2020?
5. How has the pandemic affected research activities at your site?
6. Once restrictions were lifted in your province, institution, or site, did you notice a difference in recruitment numbers? If so, what was the difference?
7. Did any of your eligible participants not yet enrolled change their decision to be in the study due to the pandemic? If so, what were the reasons they gave for changing their mind?
8. What are some reasons that parents or guardians of potential participants have given for not wanting to participate in the study?
9. Were there other reasons why people didn’t participate that have not been discussed yet?
10. Is there anything more you would like to add that has not been brought up yet?

The focus group sessions were audio and video recorded. The recorded sessions were transcribed using Trint, an artificial intelligence program to convert speech to text. This software allows for secure uploading of the saved recordings and offers a user-friendly interface for editing and polishing initial transcripts. After recordings were transcribed, two research staff (I.F. & S.H.) independently reviewed the transcriptions to ensure no errors were made. Focus group attendees’ names were not used during transcription; each attendee was assigned a unique study identification number to maintain anonymity. Once transcription was completed, the original recordings were permanently deleted from the researchers’ computers.

Field notes from the moderator (I.F.) were used to supplement the qualitative data and included observations on the focus group attendees’ nonverbal behaviours, interactions between attendees, and order of speakers.

Limited demographic data was collected from the focus group attendees which included full name, age, sex at birth, number of years in their field, and the province they worked in. The province the attendee worked in was used to see if differences existed related to differing provincial restrictions and guidelines related to COVID-19.

### Data analysis

Descriptive statistics were used to characterize the demographics of the focus group attendees. Transcripts were analysed using Colaizzi’s seven-step method of analysis, a phenomenological method which involves the examination of individual statements from focus group attendees in order to create larger themes to describe the phenomenon at hand (Colaizzi, 1978). First, research staff (I.F. & S.H.) analysed the transcripts independently, extracting significant statements. The research staff compared their individual analyses and came to an agreement on the extracted statements. Together, the researchers organized significant statements into formulated meanings, which were synthesized into larger, overarching themes. Data were analysed with the aid of NVivo, a computer software which was used to organize transcript passages once the final coding scheme was applied. Researchers (I.F. & S.H.) discussed and agreed upon the final themes describing the experience of focus group attendees involved in study recruitment. To ensure the accuracy and credibility of the results, the finalized themes were presented to all focus group attendees to ensure the themes reflect their experience.

### Methodological rigour

To ensure trustworthiness of the results, the criteria of credibility, dependability, transferability, and confirmability were used (Guba & Lincoln, 1985). Credibility, the faithful depiction of attendees’ lived experience, was achieved through member checking, as focus group attendees given the opportunity to verify the accuracy of results and challenge any misinterpretations. Transferability was achieved by using direct focus group attendees’ quotes to describe the phenomenon in detail, allowing other researchers experiencing challenges following the COVID-19 pandemic to assess the applicability of the findings to their own contexts. A detailed view of focus group attendees’ quotes organized by theme can be seen in Supplementary Table SI. Dependability, showing that findings could be repeated, was achieved through a detailed description of the research methods. Finally, confirmability was achieved by using multiple researchers to analyse findings. Two researchers (I.F. & S.H.) analysed transcripts independently before agreeing upon extracted statements to illuminate blind spots in the interpretive analysis and ensure the findings were shaped by the respondents and not researcher bias.

## Results

### Characteristics of focus group attendees

Thirteen research staff were identified by the network study Site Leads as potential focus group attendees and were invited to partake in the study. Of the 13 contacted, one was not actively involved in recruitment, one did not wish to participate in the study, two were unreachable by contact information provided, two did not respond after multiple attempts to reach them, and seven agreed to participate. All attendees were female, and age ranged from 28 to 50 years. Study Nurses, Research Coordinators, and a Family Liaison participated. Years of experience in their role ranged from 5 to 20 years. Three focus group attendees worked in British Columbia, two in Alberta, and two in Ontario (representing both Ontario network study sites).

### Focus group findings

Four main themes related to recruitment challenges experienced by research staff were identified (World Health Organization): the COVID-19 pandemic had an impact on research activity (Closure of places of non-essential business, 2020); families perform a risk-benefit analysis when deciding whether to take part in research (Rubio-San-Simón et al., 2020); a trusting relationship with clinicians is important; and (Limiting Research at CHEO, 2020) research must be flexible. The themes are presented in Table II. A complete table of significant statements extracted from focus group sessions can be found in Supplementary Table SI.

**Table II.** Themes identified, number of statements, and examples of each theme.

Theme	Number of quotes	Example (Participant number)
1. The COVID-19 pandemic had an impact on research activity	73	“*There was maybe four patients [before the COVID-19 pandemic] that were newly joining the study and we paused them and I think half of them actually we lost to follow up because of COVID [sic], because it had been paused and then they weren’t able to, to go again.” (P7)*
		*“But it’s hard now to get into the hospitals. Our hospital still has protocols to get in and strict guidelines for who can come in and visitors and stuff.” (P5)*
2. Focus group attendees found that families of children with medical complexity perform a risk-benefit assessment when deciding whether to take part in research	56	*“And even though we might think this is like a huge benefit, there’s a million other variables in each family’s life that determines whether they think that that’s true for themself in that moment in time.” (P2)*
		*“I’ve had families who have said ‘It’s going to be too long. We don’t want to continue because it’s just too involved.’” (P4)*
3. A trusting relationship with clinicians is a key factor in research recruitment	12	“ … *from my experience talking with the families that have done the PIUO study, they all were involved because they were referred by a nurse or a pediatrician or another specialist in the hospital who knew of the study and promoted it and the need or benefit for their child to them.” (P2)*
		*“I think [trust is] a huge piece…. we’re like probing into their lives every two weeks. … now you start to hear about their personal lives. So it is a trusting, right? If you don’t trust the person, why would you want to be part of something like that?” (P5)*
4. Research needs to be flexible in order to adapt to evolving contexts	36	*“ … the only reason our study was able to carry on through the pandemic is simply because we were able to expand our focus to other hospitals in the north. And that was a very creative way to see families.” (P1)*
		*“I just think that there are different expectations today to what it looks like participating in research. And so all future studies are just going to have to take that into account.” (P6)*

#### The COVID-19 pandemic had an impact on research activity

Focus group attendees across all four sites (Vancouver, Calgary, Toronto, and Ottawa) agreed that recruitment efforts and research activity were affected by the COVID-19 pandemic. Before the start of the COVID-19 pandemic, contact with potential study participants was often done in person at clinic appointments or on the unit if the child had been admitted to hospital. As one focus group attendee said:

[In] 2019, we had more than half of our participants recruited during that time in person, going to the hospital, talking to different team members. (P5)

Recruitment before the pandemic was also done through collaboration with hospital units such as Complex Care and Palliative Care, and by sending recruitment material to the hospital paediatrician list.

When the COVID-19 pandemic began, restrictions were put in place by hospitals that limited research activity. At first, there was a complete halt to all research-related activities. As one focus group attendee put it, “we were told that no one could enter the hospital” (P4). When research activity was allowed to resume, restrictions at many sites stayed in place, creating a challenging environment to recruit participants and conduct research. As one attendee put it:

But it’s hard now to get into the hospitals. Our hospital still has protocols to get in and strict guidelines for who can come in and visitors and stuff. (P5)

One focus group attendee explained that isolation policies in the hospital created significant staffing shortages, such that even if an individual wanted to participate in the study, there were no physicians available to do the medical assessment part of the study. Similarly, an attendee described that with the pandemic, many research staff were working from home instead of collaborating in an office with co-workers, consequently slowing down the administrative side of the research.

While all focus group attendees agreed that the COVID-19 pandemic had an impact on research activity, attendees from different provinces had varying experiences. In Alberta, researchers were quickly able to return to full levels of activity without having restrictions in terms of patients coming into the hospital. In British Columbia, a slow and steady approach was taken where restrictions were removed over a longer period of time. In contrast, Ontario experienced frequent lockdowns, with a cycle of restrictions being implemented and lifted. One attendee explained the challenge of approaching potential study participants during this time due to the uncertainty of when restrictions may suddenly be implemented again.

Moreover, many focus group attendees agreed that pandemic restrictions resulted in long pauses between study visits, consequently impacting study participation. As one attendee described:

There was maybe four patients [before the COVID-19 pandemic] that were newly joining the study and we paused them and I think half of them actually we lost to follow up because of COVID [sic], because it had been paused and then they weren’t able to, to go again. (P7)

Focus group attendees also described that even when restrictions were lifted at their hospital, recruitment numbers did not return to pre-pandemic numbers. One attendee explained that research activity had “really not returned to anything that it was before the pandemic, even now” (P6).

#### Families of children with medical complexity perform a risk-benefit assessment when deciding whether to take part in research

The second theme that arose from focus group discussions was about families of potential study participants and the factors that guide their decision of whether to take part in research. As focus group attendees had close contact with the families of potential study participants, they were able to share the perspective of these families. One focus group attendee explained:

And even though we might think this is like a huge benefit, there’s a million other variables in each family’s life that determines whether they think that that’s true for themself in that moment in time. (P2)

For example, the fear of exposing their child to COVID-19 was a concern for families brought to the attention of many of our focus group attendees. While this was in particular a concern at the beginning of the pandemic when not much was known about transmission of the virus, this concern for infection control has continued for these families of children with medical complexity.

Another consideration for families deciding whether to participate in research that was described to focus group attendees was the logistics of a study visit. For example, for families with more than one child, the pandemic made it particularly challenging to find caregivers to watch siblings so that a parent could attend the in-person portion of the study. Furthermore, the pandemic has impacted the convenience of participating in the study. Before the pandemic, study appointments were often scheduled around in-person medical appointments at the hospital or in clinic. However, with less in-person medical appointments available, scheduling a study appointment visit has become much less convenient. As one focus group attendee explained:

*…* I feel like it probably has affected people that maybe would be willing to come in and register, whereas they’re not coming to the hospital as much anymore…. If they don’t have to come to the hospital, then they wouldn’t sign up for something where they would have to come to the hospital. (P7)

Furthermore, since the pandemic, families have seen the benefits of virtual spaces for medical visits and are more selective about when the benefits of an in-person study visit outweigh the risks. Additionally, families consider the time commitment of the study when deciding whether to participate. A number of focus group attendees described families’ concerns with the amount of time needed to take part in the study, and the feelings of being too overwhelmed with other aspects of life to participate. As one attendee described:

I’ve had families who have said ‘It’s going to be too long. We don’t want to continue because it’s just too involved’ (P4).

Throughout the focus group discussions, many attendees agreed that in addition to time commitment being an important consideration, families must see a benefit from participating in research. Before the pandemic, one selling point for recruiting participants was that it was simply convenient to participate in the study. However, since the pandemic, participating in the study is no longer as convenient as it once was. Consequently, it is even more critical for researchers to explain the importance of the study and how the study would benefit their family. One example discussed was the value in participating in research is higher if families see it as part of their child’s health care. One attendee explained:

People have different priorities like she said and you really have to make it worthwhile for people now to be involved. Because they just… Time is more precious for, now than it was before. And people aren’t willing to spend the time unless they see a direct benefit to them… (P7)

#### A trusting relationship with clinicians is a key factor in research recruitment

Focus group discussions revealed relationships being an important factor in research recruitment. Attendees identified limited face-to-face interactions and relationship building due to the COVID-19 pandemic as barriers to recruitment. They described more success speaking to nurses and physicians in the hospital who could then refer patients to the study, rather than sending emails that often went unanswered. As one focus group attendee expressed:

*…* during the pandemic, not being able to create as many relationships in person with families or with other colleagues that could then refer those families… when you’re working from site and working in the hospitals, you can have those conversations and build those relationships more… so when less people are in the hospitals and in the clinic spaces, there’s less opportunity for those relationships and then to find families”. (P2)

Moreover, attendees identified medical referral as being the most successful method of recruitment because of the trusting relationship potential study participants have with their clinicians. It was discussed that many families got involved with the study because they were referred by a clinician that they trust. Furthermore, once involved in the study, participants’ families valued the relationship they had with the research nurse. Focus group attendees agreed that many families appreciated the frequent phone calls with the study nurse and that a trusting relationship was built through these recurrent connections. As one attendee explained:

And knowing that the people, the nurses that were speaking with them every couple weeks and helping them along through the study, weren’t just doing it as researchers, but it was people who understood their family’s situation and understood the complexity of their child … they felt validated in their experience or their child’s pain was real and acknowledged. (P2)

#### Research needs to be flexible in order to adapt to evolving contexts

A common frustration shared by the focus group attendees was the lack of flexibility the study had in terms of adapting to the COVID-19 pandemic restrictions. When the COVID-19 pandemic began, research staff had to engage in creative strategies to adapt to circumstances resulting from the pandemic. Before the start of the pandemic, contact with potential study participants was often done in person at clinic appointments or at the hospital. When the pandemic began, hospital restrictions made this strategy challenging. As such, creative recruitment strategies were adapted such as using social media to reach families, recruiting participants through the emergency department, and expanding recruitment to different areas of the province. As one focus group attendee described:

*…* the only reason our study was able to carry on through the pandemic is simply because we were able to expand our focus to other hospitals in the north. And that was a very creative way to see families. (P1)

Furthermore, different sites used novel strategies to conduct study visits. One site transitioned to virtual assessments when appropriate, and another site borrowed spaces in private clinics when hospitals were not as accessible.

However, adapting new strategies was not always simple. Focus group attendees described the challenges of being creative in research where there are a strict set of rules and guidelines to be followed. For example, one attendee described the challenges of obtaining Research Ethics Board approval to transition to electronic consent forms:

Just to submit to the REB to get e-consent was… It took me so long, because they kept coming back with wording and this and that. And I was thinking to myself, if I wasn’t persistent, I would give up… it shouldn’t be this hard to try and pivot to make something work… It’s difficult to be creative, even in a small way with research. (P5)

When discussing adaptability and evolving contexts, attendees also described advances in healthcare and how changes in the medical field may have impacted study enrolment. It was discussed that a potential reason for low study enrolment is because families and clinicians alike are more aware and better at managing pain and irritability in children meeting the PIUO study criteria. Furthermore, one focus group attendee described the impact of medical education and how new graduates are perhaps more empathetic towards pain. Another attendee explained:

I think the ten years that we have tried to create capacity both in complex care and in palliative care, we’ve really come a long way in terms of treating symptoms…. the capacity to treat and see these kids clinically and also the realization that pain and irritability is a symptom that is worth focusing on has come a long way in that time. (P1)

Finally, focus group attendees discussed how the pandemic has shifted potential study participants’ ideas of what it should be like to participate in research. With advances in technology and connecting meaningfully online, participants don’t necessarily want to come in for a traditional in-person study visit anymore and research must adapt in order to meet these new expectations. As one focus group attendee explained:

I just think that there are different expectations today to what it looks like participating in research. And so all future studies are just going to have to take that into account. (P6)

## Discussion

The COVID-19 pandemic has had an unprecedented impact on the lives of citizens around the world. To help prevent the spread of the COVID-19 virus, strict public health guidelines were enacted within local hospitals and affected research centres, such as pausing in-person recruitment efforts and visits with study participants (Limiting Research at CHEO, 2020; Rubio-San-Simón et al., 2020). This qualitative study provides insight into the recruitment challenges experienced by research staff from a paediatric network study, where the recruitment rate dropped following the start of the pandemic. Focus group attendees identified several recruitment challenges following the onset of the COVID-19 pandemic as well as other non-COVID-19 related factors that impacted study recruitment. These findings can be applied broadly to research groups encountering recruitment challenges because of the COVID-19 pandemic or otherwise. Insights gained, such as employing electronic signatures for consent and the need for flexible research protocols, are lessons that can inform future research endeavours across diverse fields.

Focus group attendees agreed that recruitment efforts and research activity were affected by the COVID-19 pandemic. Some of these factors included limited opportunities for in-person recruitment, families’ concerns over infection control, and long study pauses due to restrictions. Studies from across the globe have had similar findings of significant impacts of the COVID-19 pandemic on research activity (Chowdhury et al., 2021; Radtke et al., 2021; Rubio-San-Simón et al., 2021). One study evaluating the impact of the pandemic on clinical trial activity in paediatric oncology found a 75% reduction in recruitment rate compared to the monthly average in 2019 (Rubio-San-Simón et al., 2021). Another study surveyed over 1000 individuals with rheumatoid arthritis and determined that respondents were less willing to consider participation in rheumatology research studies while COVID-19 was circulating in the community (Mirza et al., 2022).

Furthermore, focus group attendees suggested that challenges presented by the COVID-19 pandemic have had a particular impact on families of children with medical complexity (CMC). CMC have special healthcare needs with chronic conditions resulting in frequent healthcare interactions across multiple settings (Cohen et al., 2011). A concern for families of potential study participants that was described to research staff was that their child with medical fragility would have poor health outcomes if infected with COVID-19. A recent study looking at underlying medical conditions associated with severe COVID-19 illness in children revealed that neurodevelopmental disorders as well as medical complexity were associated with a higher risk of severe illness (Kompaniyets et al., 2021). Moreover, due to pandemic restrictions, CMC experienced disruptions in therapy and rehabilitative services, home care and respite services, and medical care (Diskin et al., 2022). These disruptions have exacerbated the psychological stress experienced by caregivers of CMC (von Schulz et al., 2022), making these families less likely to participate in research (Tripp et al., 2022). Future researchers must demonstrate flexibility to newly emerging contexts which may impact research participants and may need to tailor their approach based on the population being studied.

In addition to infection control being a recruitment challenge posed by the COVID-19 pandemic, restrictions on in-person research visits caused long pauses between study visits such that some participants fell outside of the study protocol timeline. One way to mitigate the negative effects of future research pauses would be to have contingency plans built into research protocols that allow for study pauses or a process for rapid ethics board reviewal of protocol modifications. For research to be successful in a world where the probability of novel disease outbreaks is expected to increase in the future (Marani et al., 2021), it must be flexible and adaptable to different situations. For example, a recent study looking at solutions to maintaining research networks during the pandemic described the importance of building adaptable frameworks that ensure the protocol yet adjust to the unique circumstances that are impacting the study (Santillan et al., 2022).

Despite restrictions being lifted at hospitals, recruitment numbers for this paediatric network study did not return to pre-pandemic levels. Since the COVID-19 pandemic, families of potential participants have had different ideas about what research should look like. With the transition to virtual platforms for healthcare visits and other services, families have seen the benefits and convenience of virtual spaces that could be incorporated into research. The rapid expansion of telemedicine triggered by the COVID-19 pandemic provides an opportunity to expand access to research. Travel time and distance are common reasons why potential study participants decline to participate in research (Deng et al., 2022). Virtual services may increase access for individuals in rural areas (McCormick et al., 2022) as well as reduce travel burdens and costs (Santillan et al., 2022). However, there are limitations to virtual visits, such as performing a comprehensive physical examination, access to investigations, technical issues, and data privacy (Khoshrounejad et al., 2021). Future research should explore the options of semi-virtual or hybrid studies to balance convenience and accessibility for study participants with the feasibility of online visits.

In addition to many recruitment challenges caused by the COVID-19 pandemic, this study identified other non-COVID-19 related factors that impacted study recruitment. These factors are related to gaps in the research-to-practice continuum, the translation of discoveries generated by basic biomedical research to clinical practice (Nieva et al., 2005). Specifically, this study identified the challenges of conducting research during a time period when clinical practices change. Focus group attendees wondered whether reduced study enrolment was due to families and clinicians being more aware of and better at managing pain and irritability in children meeting the PIUO study criteria. For example, two retrospective studies have supported the use of gabapentin, a neuropathic pain agent used in adults (Mu et al., 2017), for pain management in children with severe neurological impairment (Collins et al., 2019; Hauer & Solodiuk, 2015). The rising use of gabapentin in children in recent years may be contributing to better pain management in children who otherwise would have met criteria for the PIUO study.

Focus group attendees described potential participants as being more likely to enrol in the study if families saw the research as part of the child’s healthcare. Moreover, physician referrals were described as the most effective method of study recruitment. These speak to the value of a trusting relationship between potential participants and their clinicians for research recruitment. It would be prudent for health care providers and families to be current in available research that could be beneficial for the child.

## Limitations

This study used purposeful sampling of past and present research staff working on a network study. Given the pre-existing professional relationships of research staff with each other, there was a risk of social desirability bias during the focus group sessions. This bias was mitigated by use of a moderator who had no pre-existing relationship with the participants to encourage open and honest discussion. This study has a small sample size with uneven representation across the four study sites, with a greater proportion of participants having worked at one site. However, all study sites were represented in the focus group sessions and the different representations can be attributed to some study sites being larger than others. One focus group was smaller in size than the other, which may have limited the dynamic interaction between attendees in the smaller group. However, when analysing the focus group transcripts, many similar themes were evident in the two groups. Limitations also arise from the use of a virtual platform for conducting focus group sessions. While all participants had their cameras on to encourage the use body language and gestures, engagement can be limited on online platforms (Canada’s Strategy for Patient-Oriented Research, 2011) and attendees may have had different levels of comfort participating in a virtual setting. Also, potential for technical difficulties existed. Importantly, using an online platform allowed participants from different provinces to meet safely, following infection control guidelines in place at the time of the study. Finally, this study did not include families of potential participants as focus group attendees, so recruitment challenges from the perspective of families were obtained indirectly from research staff. This limitation was partly mitigated by the fact that research staff were in direct contact with families of potential participants and when relevant, explicitly asked families their reasons for not wanting to participate in the study.

## Conclusions

In this qualitative study, several recruitment challenges were identified by research staff. These included factors related to the COVID-19 pandemic as well as other non-COVID-19 related factors. Understanding and addressing these research challenges will mitigate the negative impacts on health outcomes that can occur when research studies fail to meet enrolment targets. Researchers must be flexible in the face of evolving contexts such as the COVID-19 pandemic in order to continue research that will positively impact patients’ lives.

## Supplementary Material

Supplementary Table 1 .docx
