# Supplementary material for: Research staff’s experiences of how the COVID-19 pandemic impacted recruitment for a paediatric network study
Source: Int J Qual Stud Health Well-being. 2024 Oct 22;19(1):2419158. doi: 10.1080/17482631.2024.2419158 (PMC11497574; doi:10.1080/17482631.2024.2419158)
Supplement: Supplementary Table 1 .docx [file ZQHW_A_2419158_SM6554.docx]

**Supplementary Table 1. Significant statements extracted from focus group sessions arranged into themes.**

Theme 1: The COVID-19 pandemic had an impact on research activity

| *“…we had a number of clinics that were helping us with recruitment before the pandemic.” (P1)* |
| --- |
| *“It was quite easy to go and visit kids in the clinics…” (P1)* |
| *“…we would make first contact with them, sometimes actually on the unit if they had been admitted, or try and time it with an appointment…” (P1)* |
| *“…there seemed to be a good number initially and then less and less as time has gone on.” (P2)* |
| *“…because I'm obviously not in those circles, it was like harder for me to get into the parent groups and sites, of course.” (P3)* |
| *“…we were so lucky with the group member we had who had that reach as well.” (P3)* |
| *“But for me, I reached out to a lot of like clinics.” (P3)* |
| *“Just because of COVID there was a lot of impact [on recruitment efforts]…” (P3)* |
| *“… I would say the best recruitment for our end was like physicians referring their patients over to the physicians that were the PIs for our study.” (P3)* |
| *“…the doctor that I work with, she also works in complex care, which is where we've gotten the majority of our patients from.” (P4)* |
| *“So it started off quite well at the beginning.”* |
| *“But the patients just aren’t there... we’ve put out blitz... and they’re just not coming through the door.” (P4)* |
| *“2019, we had more than half of our participants recruited during that time in person, going to the hospital, talking to different team members.” (P5)* |
| *“… most of them came from the palliative care center that I was working at at the time.” (P5)* |
| *“Since [the pandemic started], we’ve had almost no one in our site, even though we’ve actively tried to recruit..., we haven’t had many people interested.” (P5)* |
| *“…recruitment has been very, very low. I don't have details. It was after a closure and we reopened.” (P4)* |
| *“…there wasn't the interest as what there was when we initially started this study. So whether that's, like I'm assuming it's because of the pandemic or was it that we gave you all the names of the people that we know initially and we just don't have any other names to provide you with.” (P4)* |
| *“We had a huge drop after the restrictions finally kind of loosened a bit in [my province]. But I also don't know if it's because of that or because we ran out of people to see.” (P5)* |
| *“... maybe some of our potential participants ended up going to [a different site], right. Instead of our site.” (P5)* |
| *“I did a blitz at the beginning of the study when I started, I did a blitz shortly after, I think the first or second time restrictions were lifted. The response I had the first time was a bunch of nurses who work in these units sending me names and referrals. The second time I got zero emails back.” (P5)* |
| *“I don't know if people were just fatigued, didn't have names left, maybe they don't work in those settings anymore, those units. There's so many factors, it's kind of hard to pinpoint exactly what caused it, but we definitely have a lower number after the pandemic and the restrictions than before.” (P5)* |
| *“…I can tell you they definitely didn't go up.” (P1)* |
| *“And maybe their awareness of how much the study would involve for themselves to participate, the time and and commitment level, rather than just like a quick social media post that might not give them that same information.” (P2)* |
| *“… it really does make a difference how these kids come to you. And so right from the very start, you know, whether this study is going to be successful in enrolling, it's from that very first person or very first way that you hear about the study.” (P1)* |
| *“…the real backbone of our solid families who show up, who consider this a medical intervention, comes from the medical referral…” (P1)* |
| *“... initial way to recruit was they sent out some recruitment material through the hospital pediatrician list, as well as the neuro motor database.” (P7)* |
| *“I think there has been. One, two... I think four since COVID started. But then we haven't had that. We had like a huge break. That we hadn't had anyone. Since... Almost a year, I think, since we had recruited.” (P7)* |
| *“… the recruitment efforts were sort of...um... Great. And they were both, you know, both sending out letters, word of mouth, etc., etc.. We had a lot of response…” (P6)* |
| *“…It was really just with the like when the pandemic hit, it was like - no one. And then slow since then. But we averaged I think we averaged about like 3 to 4 recruits a month in the... Before the pandemic. And then went down to zero. And then it became like more like 1 to 2.” (P6)* |
| *“... The recruitment itself. It also came to a halt, obviously, as the pandemic hit, because nobody you know, we didn't see anybody anywhere. And then it's been really hard getting back.” (P6)* |
| *“I don't believe, from my memory and from what I can find in the notes here, that anyone changed their mind [about participating in study before being enrolled].” (P7)* |
| *“I don't think we had anybody change their mind [about enrolling]…. I think it was more that we like never got talking to some people in the first place. Like they were like, ‘no, not interested in research,’ but for the ones that we'd already talked to, They're... I mean they are usually quite keen to have that, you know, to get the services that they get through the study. So I don't think we had anybody change their mind as such.” (P6)* |
| *“I can't see any that were eligible, but didn't want to be part of it.” (P7)* |
| *“But unfortunately, that [more social media recruitment] didn't actually bring more people in at the time.” (P7)* |
| *“…we had two or three pauses and then we had some appointments delayed because at the time all research activity was canceled…” (P3)* |
| *“PIUO was on hold.” (P3)* |
| *“…once restrictions loosened up a bit, we did some of the physical exams on site.” (P3)* |
| *“…due to the pandemic, we were told no one could enter the hospital.” (P4)* |
| *“We did lose one patient to COVID because we had to pause their medical assessment for so long…” (P4)* |
| *“And then we had quite - I can't remember if it was two or three or I don't know how many times where we had like no one could come in to the hospital.” (P4)* |
| *“...two that we lost because... we had to pause and then they missed the MD assessment and then never got back to it.” (P5)* |
| *“We were just on hold, like in [my province], for sure.” (P3)* |
| *“…and I was on a couple of studies at the time and all but one was basically like canceled, not allowed and not started kind of thing.” (P3)* |
| *“…we weren't able to have any like research related visits.” (P3)* |
| *“…later when we did, we did have to reach out to the like managers to let them know that this person is going to be coming in for research related reasons. And then we had to fill out like a separate form to allow more than one person in.” (P3)* |
| *“…the hospital, yeah, like at the very beginning especially, it was all canceled. Nobody was really allowed to continue the research activity except for like very few niche studies.” (P3)* |
| *“Research activity was also put on hold for a long time…” (P5)* |
| *“And then even when we were allowed to restart our activity, limited amount of people who could come into the actual site.” (P5)* |
| *“…our center as well didn't go back to a full level activity… had less people and less opportunity to catch these people in-house. So the restrictions really made it difficult..., even if they were interested. They're like, ‘This sounds great, but all these factors are playing into me not being able to get there.’” (P5)* |
| *“We all got locked out right at the beginning.” (P1)* |
| *“…we were locked out of our offices and out of our research clinic space, which is where we do our assessments for, three full months before we were able to submit a special exemption.” (P1)* |
| *“We were then able to get access to our clinical files, which we hadn't had at home.” (P1)* |
| *“…that roller coaster of full restriction and then let's lift some so that everyone's trying to ramp back up. And then it was lockdown again and then out of lockdown. It just made it hard to try and even actively approach people because you're like when they approach you, if you end up on the waitlist, I don't know if in that amount of time that goes by, I'll be able to see you...” (P5)* |
| *“…you guys had an even worse roller coaster than anybody else … and a lot of this stems back to the provincial government, right? We had our [provincial public health officer] slow and steady. And so we didn't really have to roll back anything back and forth.... definitely the provincial governments were having some influence there.” (P1)* |
| *“…for research purposes kind of made it more difficult because other parts of the province were able to do a bit more than us.” (P5)* |
| *“Our numbers were always kind of tough just because I think like the big team kind of started right at the beginning of the pandemic.... our recruitment did go up certainly once restrictions lifted. But I don't know if that was because of restrictions lifted or it was just the timing of the study that that's when we were actively recruiting more.” (P3)* |
| *“… because of COVID, a lot of times custody issues kind of got confusing too, because it's like now you're in multiple households and things like that.... I think potentially infection control, potentially consent was the issue.” (P3)* |
| *“But it's hard now to get into the hospitals. Our hospital still has protocols to get in and strict guidelines for who can come in and visitors and stuff.” (P5)* |
| *“It actually often is our own institutions that set up the barriers to research. Unfortunately.” (P1)* |
| *“We were so short staffed, we didn't have doctors. And even if I did have patients that needed a medical assessment, even if I could have brought them in, I wouldn't have had anyone to see them because we were running like basically like a skeleton crew, because everyone was- we were still- even if you were a contact, you were isolated for ten days.” (P4)* |
| *“There was maybe four patients that were newly joining the study and we paused them and I think half of them actually we lost to follow up because of COVID, because it had been paused and then they weren't able to, to go again.” (P7)* |
| *“We closed down completely. I'm really bad with memory in terms of actual timing, but so we stopped everything. We put the study on pause for six to eight... Six months maybe?” (P7)* |
| *“We were able to bring patients in. We were able to do everything the same as before. We didn't have any restrictions in in terms of patients coming into the hospital.” (P7)* |
| *“…I would say things like have really not returned to anything that it was before the pandemic, even now.” (P6)* |
| *“…when the pandemic hit, but there was, you know, we were all working from home and since there was no, you know, we couldn't go in and see people then it would like effectively we closed down for a little bit.” (P6)* |
| *“…we used to work in an office together and be able to, you know, liaise with each other and talk about things as we're sitting there next to each other. And that really hasn't happened since the pandemic... it has slowed down everything…” (P6)* |
| *“People don't have access in the same way to our actual paper files all the time, at the same time.” (P6)* |
| *“… And it's not directly recruitment, but it does, you know, this is all the backup and the support for recruitment and that has been impacted greatly and is still impacted.”  (P6)* |
| *“I have noticed a huge change in that they don't do in-person appointments as much.” (P7)* |
| *“That's been hard for us because no, it wasn't sort of like, "okay, lifting restrictions. Now we're back in the hospital." Really it has not changed that much since. Like the... Unwillingness, you know, on good grounds by our families to come to the hospital. It continues to be there.” (P6)* |
| *“For some, it's been sort of like when restrictions were lifted. ‘Okay, great. We can be seen’”. (P6)* |
| *“I think in general, in [Province], people weren't as hesitant to come back to the hospital when restrictions were lifted because it was very much like everything was closed and then everything was open.... They weren't as hesitant to come to the hospital, if they if they needed to.” (P7)* |
| *“…I think that the pandemic is one thing and it's sort of like, you know... A big barrier in itself.” (P6)* |

Theme 2: Focus group attendees found that families of children with medical complexity perform a risk-benefit assessment when deciding whether to take part in research

| *“…they have to use wheelchairs and stuff. So and then like some parents had multiple kids. So it was just really difficult for them to, like either leave a sibling at home or be able to physically carry both kids inside. So in order to have like another caregiver come in, it was like a lot of paperwork that we had to provide…” (P3)* |
| --- |
| *“…parents do a risk benefit analysis on every activity that they do with their children. And research is no different. … and maybe virtual is a better option for our family.” (P2)* |
| *“I've had people just stop answering the phone when I call them.” (P4)* |
| *“…I also had a family who had a new baby when they were enrolled on the study and then got a bad diagnosis for the baby - like midway through the study. Like there was too much going on at home, requested to be removed from the study.” (P4)* |
| *“… it was incredible how well she was able to avoid talking to me on the phone.” (P1)* |
| *“But I also had families who seemed really keen and interested, and then I would send off a consent form and then I would never be able to reach them again.” (P5)* |
| *“And even though we might think this is like a huge benefit, there's a million other variables in each family's life that determines whether they think that that's true for themself in that moment in time.” (P2)* |
| *“…the value in it is probably higher if they see it as part of the child health care system, as opposed to perhaps just another survey they found online.” (P1)* |
| *“... that whole protest, because even though it wasn't necessarily right next to the hospital, it blocked a whole core of the downtown. People were scared about going out. Being confronted with some of these people.” (P5)* |
| *“…we were affected by the protests as well because there's like five hospitals in a row and they were protesting at [the government building], which is just up the road. So they actually like blockaded the whole area to protect the hospitals. And that was for weeks. Like, maybe even like a month. Like a long time. So that did affect us.” (P4)* |
| *“And I would say we've more had the other way around, like not so much people giving reasons why they don't want to do it, but more people wanting to be eligible when they're not.” (P6)* |
| *“So the two patients that we lost. There was no reason, they just couldn't get a hold of them after.” (P7)* |
| *“You know, a good challenge that we really have to tell people now why this research is important and why. And also think about within the research, so what exactly are we giving people, right?” (P6)* |
| *“People have different priorities like like she said and you really have to make it worthwhile for people now to be involved. Because they just... Time is more more precious for, now than it was before. And people aren't willing to spend the time unless they see a direct benefit to them, which is tricky with the study because we even say that there may not be a direct benefit to them. So I think that that mindset has just... Like... That before that was known, but maybe people were still okay with doing it for the better, the goodness of the the whatever you call it, that of the whole population. Whereas now they they may not be as willing to do it unless they know they're going to have a direct benefit to them.” (P7)* |
| *“… regarding COVID specifically, it was like parents being worried about entering the hospital because it's like one more appointment that the kid could be exposed to COVID.” (P3)* |
| *“And then the pandemic started … didn't want to come into the hospital for fear of exposing their child, potentially to COVID.”  (P5)* |
| *“… these children are so complex and so frail, the parents just didn't want to come in for a study, right? To potentially expose their child to COVID. And especially at the beginning when we didn't know all the information we have now… It really turned a lot of people off wanting to come in.” (P5)* |
| *“… we had one family who immediately said no… It was absolutely infection control.” (P1)* |
| *“…I can think of one family who didn't go to the hospital and, same thing, infection control… ‘no, they are still very COVID cautious. I wouldn't even reach out - don't bother reaching out to them.’” (P4)* |
| *“…we did have two kids who were from the same family, enrolled, and they started before the pandemic. Then the pandemic hit… they didn't feel comfortable coming in for infection control.” (P5)* |
| *“… they do not want to schedule 1 million appointments at the same time because they don't want to see a million people in one day.” (P6)* |
| *“…it's hard when parents have more than one kid and have no one to watch your child, especially during the pandemic. You know, you can't really easily find someone to come watch your child. So added layer of difficulty.” (P5)* |
| *“…their risk benefit analysis is like constantly shifting and the pandemic has thrown that all up in the air, not just because of the COVID being a risk, but also the benefits of virtual spaces and the benefits of being more picky and choosy about how often they go to the hospital for different things.” (P2)* |
| *“... we have families for whom the pandemic hasn't ended.... There are families with whom those restrictions will be in place for a long time.” (P1)* |
| *“…many of these families are looking at a lot more than just what public health is saying.... they're going to approach coming in for something, whether the hospital says it's safe or not a lot differently than what's being told to them.” (P2)* |
| *“But I know a few parents now that I think about it did say like at least now we have a physician we can speak to because a lot of pediatrician offices were closed..... it was just like no access.... And on more than one occasion, we did have people come to us with like alternative concerns...” (P3)* |
| *“…I think recruitment potentially went up because of the potential access.” (P3)* |
| *“We don't have any families who say they don't want to participate.... By the time we get them on the phone, we are kind of their, you know, bastion of last resort.... But again, you have to jump through so many hoops to finally get to the research nurse. It's by the time they get me on the phone, they're almost always already bought in.” (P1)* |
| *“I wonder if [patient’s not follow up is] related to what was brought up earlier with access to care and how during the pandemic they couldn't even get in to see their regular doctor, right? And here's a study who's offering literally a barrage of tests if needed.... And so this may have been a way for parents to get some answers to things that they've been waiting for and not done nefariously at all.” (P5)* |
| *“I would say a shift that I would have noticed is that people that wanted to be seen, would be happy, that now it could happen.”  (P6)* |
| *“… every family would get very primal and it's like survival or that was sort of, you know, the pandemic hit and it's something new for everyone. And so everybody would have that... Gut reaction to do survival. And I think in that... In that state, you're not really thinking, "oh, let me do your research." You know, you're like, "no, that's like that's just not a priority because it's not. It's what... Not, not what I need to do to survive right now. Me and my family.”” (P6)* |
| *“…what do I get for helping you during the pandemic?” (P6)* |
| *“It's almost like a cultural shift [that this pandemic has caused].” (M)* |
| *“…coordinating those visits together that it's like it's such a hassle to get in.” (P4)* |
| *“I've had families who I've tried to recruit who said they didn't want to come down to the hospital... They're already at the hospital often enough. They don't want to come for yet another appointment...” (P4)* |
| *“…perhaps the way the study was formatted, was created. There could have been maybe some room to put a bit less burden on the families.” (P5)* |
| *“… they they would just be coming in and scheduling wasn't really a huge issue. And I mean, it's always like a bit of a logistics issue, but it wasn't a big issue because they would be coming to the hospital at some point from all over the province. And now that's really not the case, like since the pandemic hit.” (P6)* |
| *“…I feel like it's probably has affected people that maybe would be willing to come in and register, whereas they're not coming to the hospital as much anymore.... If they don't have to come to the hospital, then they wouldn't sign up for something where they would have to come to the hospital.” (P7)* |
| *“’…if I don't have to go to the hospital, that is a big hassle. And then I don't want to.’” (P6)* |
| *“… but mostly people that was sort of like a bonus. You can do this study while you're here anyways, so it's not a big deal. But I mean, it's still it's time to see us and so on. But. But yeah, I think that that idea is not really that much there anymore because there isn't that those other appointments to tag on too.” (P6)* |
| *“…that sort of like ease of doing it together with all the million other appointments that's that's just gone. And I'm not sure it's coming back.” (P6)* |
| *“… my hunch is that people, the ones we have lost to follow up, it's a matter of availability really, or accessibility. That it's not... It's just too much for that family to make an appointment happen or sometimes it's not even, you know, the appointment might have happened, but it's some follow up test or something like that.” (P6)* |
| *“… there's different expectations to how convenient it is to do research. And it has to be convenient for people like they have to... It has to be accessible on their mobile devices... It has... you know, there can't be like a huge amount of travel time. And of course, it depends a little bit, like also people that have like kids with severe complexity, like they're going to come in and see doctors.” (P6)* |
| *“Even I've noticed like this one that I've had post pandemic … typically before we are doing the follow up with by telephone and I would occasionally send an email, but it was more telephone, whereas she was like, ‘Just send them all to me by email. It's just way easier than having to coordinate a time to do a phone call…’” (P7)* |
| *“... ‘Oh no, I don’t really know that I want to have to come in when I’m already in the hospital for so many visits.’ … ‘Oh, that’s going to be such a long day because we’re going to see all these appointments’ and it makes for a very long day for the families as well.” (P4)* |
| *“We just had some parents overwhelmed, I think, with everything. So they were like, I would love a pause or I would love to take some time off, or I would just like to not continue it's just too much, too many appointments.” (P3)* |
| *“Both of the people that I didn't get consent from, once I spoke to them and discussed the study, didn't want to because they weren't interested anymore. Once they heard more about the study in depth. And I think it's to do with the fact that you kind of go through, every two weeks, I'm going to call you and we're going to do this survey and then you're going to do a longer survey at point one, two and three. And you have to come in one time for an MD assessment. It's a lot.” (P5)* |
| *“It's a lot. It's a lot for some parents.” (P5)* |
| *“...other ones were just feeling overwhelmed and thought that was just way too much for them to take on with everything else they were doing.” (P5)* |
| *“I actually have had quite a few families... lost to follow up. So some reasons are being placed on the waitlist arm.” (P4)* |
| *“I've had families who have said ‘it's going to be too long. We don't want to continue because it's it's just too involved.’” (P4)* |
| *“ Some families have made comments on that as well, that both the time aspect of it, but also like just the mental load of it.” (P2)* |
| *“… the study was created. Like how long ago? So back then, the patients that we're looking for just seemed to be in abundance, right? And now they're not.” (P5)* |
| *“It's more that they don't have time.” (P6)* |
| *“…I think people just aren't as willing anymore to do anything. Outside of, like... yeah, it's just time. It's just more time consuming and people just aren't as willing to participate anymore.” (P7)* |

Theme 3: A trusting relationship with clinicians is a key factor in research recruitment

| *“There was one parent who was so happy to talk to me every two weeks. It was like we were friends. We would chat for so long. It was her connection to the world, right?” (P5)* |
| --- |
| *“Most families, all families that I've talked to, got involved because they were referred by a clinician that they trust. And I think that's a hard thing about this online recruitment. It gets the word out and it gets people aware of something, but if they don't know the clinicians or the researchers that they're going to be working with or if somebody that they know and trust wasn't the person backing it initially, I think they're less likely to follow up or to get involved, perhaps.... from my experience talking with the families that have done the PIUO study, they all were involved because they were referred by a nurse or a pediatrician or another specialist in the hospital who knew of the study and promoted it and the need or benefit for their child to them.” (P2)* |
| *“I think [trust is] a huge piece.... we're like probing into their lives every two weeks.…now you start to hear about their personal lives. So it is a trusting, right? If you don't trust the person, why would you want to be part of something like that?” (P5)* |
| *“I did have one woman when we were finished our six months. She said she was going to be sorry to not speak with me anymore because she said ‘It feels like I'm losing that connection, you know so much about my personal life and my family's life. It feels like you're losing a friend almost.’” (P5)* |
| *“…you are digging into their life. And this was during the pandemic, and mom had no one else to talk to... but I was someone for her to talk to every two weeks.” (P4)* |
| *“…definitely one that said this explicitly, but maybe more, that the worst part of the study was when it ended. Because their connection to the nurse ended.” (P2)* |
| *“And I think the trust part made a huge difference for them.” (P2)* |
| *“And knowing that the people, the nurses that were speaking with them every couple weeks and helping them along through the study, weren't just doing it as researchers, but it was people who understood their family's situation and understood the complexity of their child…they felt validated in their experience or their child's pain was real and acknowledged.” (P2)* |
| *“I think the the relationship part of it has been a big part of this study.” (P2)* |
| *“… during the pandemic, not being able to create as many relationships in person with families or with other colleagues that could then refer those families... when you're working from site and working in the hospitals, you can have those conversations and build those relationships more, which is just like a spiral effect, I think. And then reaching families. And so when less people are in the hospitals and in the clinic spaces, there's less opportunity for those relationships and then to find families.” (P2)* |
| *“…I had more success going into the hospital, … and going to the units and speaking to the nurses directly or the physicians and just have a chance to explain to them the study... It's harder to brush off someone who's right in front of you and you're talking to, then getting an email, which if you're in a rush... and then you can easily ignore that email...” (P5)* |
| *“…people are more empathetic when parents are missing a medication or parents are not able to bring the kids in… the empathy aspect is definitely a big takeaway. That'll hopefully help with maybe recruitment too?” (P3)* |

Theme 4: Research needs to be flexible in order to adapt to evolving contexts

| *“…we really moved everything online after that [pandemic].” (P1)* |
| --- |
| *“…I'm in a few different Facebook groups that are specific to that, mostly in [one of the study provinces], but I've also tried to extend that reach to [other cities].” (P2)* |
| *“…we did go online…” (P3)* |
| *“There is maybe three or four that we did the MD assessments virtually…” (P4)* |
| *“I imagine that of all of the people that we recruited since the pandemic, only one or two have been in [the city centre]. And we only continue with enrollment simply because we're able to expand our reach up North.” (P1)* |
| *"And we tried to accommodate, like what if we do the history part on the phone which usually takes the longest, right. Do that over the phone and then we can visit you for like a quick 15-20 minute assessment. We could even drive, like the [Site Lead] and I even said we would drive to you and go to your house and wear our PPE.” (P5)* |
| *“…the only reason our study was able to carry on through the pandemic is simply because we were able to expand our focus to other hospitals in the north. And that was a very creative way to see families. I know in [a city] they moved to telehealth and that was another creative way.” (P1)* |
| *“I think the real struggle is that health care systems are not creative…that's a real struggle when you have such a major shift in our entire, you know, not just Canadian, but global way of doing things, that you have a health care system that is not really great at adapting. And a subset of that, which is a research, you know, set of rules and guidelines, which is even less able to adapt.” (P1)* |
| *“Just to submit to the REB to get e-consent was... It took me so long, because they kept coming back with wording and this and that. And I was thinking to myself, if I wasn't persistent, I would give up... ... it shouldn't be this hard to try and pivot to make something work... It's difficult to be creative, even in a small way with research.” (P5)* |
| *“Like in our area, [a different province] is like a stone throw away, right? And we can't access all those patients.... And I can't get over there because the [site lead] doesn't have the licensing for that province. Yet, we're in the same country, so that's made it difficult for us to do it on our end too. Just recruiting and seeing patients and getting them on board.” (P5)* |
| *“…I wonder how much too that parents are more aware and are asking different questions that they would have asked ten years ago, because we all communicate with each other online.... We find somebody else who's been through something similar, and then we learn what we then should ask our own specialists and clinicians to help our child… they bring that knowledge and curiosity into their clinics as well, and maybe are finding things quicker” (P2)* |
| *“I mean, every parent I've spoken to, when they say their child has pain, it's like, "oh, so what did you do to try and help them?" And then the list of things that they've done already, right? It's exhaustive… But I'm sure they're based on their own previous experiences talking to other people and talking to their doctors a bit more openly and freely, and not just going with like, ‘Oh, the doctor said this so I'm going to do that’, like questioning and asking more questions.... Like just being a bit more involved.”” (P5)* |
| *“... there was a little bit of social media recruitment, but not a huge social media recruitment at the beginning... within the last six months... more of a social media push.” (P7)* |
| *“We just started through the emergency at the children's hospital.” (P7)* |
| *“We had to completely rethink our strategy. And now we go every single participant that is outside of [City], we go see them. And it's like $1,000 every time.” (P6)* |
| *“…it's a big it's a big cost, and it's a big you know, it's a lot of planning as well. And a lot harder because, you know, our nurses and I have gone too. We can go on a trip somewhere, but it can be hard to get a doctor to find a day that they can rip out of their calendar to go to [city up north] or go to [city up north] or somewhere else.” (P6)* |
| *“Like we've seen people in private clinics instead, like just borrowing a space so they could come there.” (P6)* |
| *“It [pandemic] has shifted potential participants' idea of what it would, could and should be like to participate in research. And so what it what we used to do is not really what people want to do anymore.” (P6)* |
| *“But I think it is like a challenge because our study as it is now, is set up in a different way, like it's set up more traditionally.” (P6)* |
| *“And I just think that there are different expectations today to what it looks like participating in research. And so all future studies are just going to have to take that into account.” (P6)* |
| *“Like we can change it so much, but there are things that are the rigid the way they were to begin with.” (P6)* |
| *“Changed was we did a lot more of the recruitment on the like social media side of things because that has just changed a lot since post-pandemic.” (P7)* |
| *“…it's sort of like a challenge to researchers to really show... We really have to show... Potential participants or people in general, what you know, what the worth of our research is and why it's important to do it.” (P6)* |
| *“...I think pre-pandemic you might be able to... A selling point for research would be like, "oh, it's really not very bothersome. It only takes a little bit of time." But that's like not really a selling point any more. People are willing to spend the time, but it needs to be worth it…” (P6)* |
| *“Or they're being treated appropriately now. And so they don't need the study.” (P5)* |
| *“I know that ten years ago when we were doing the pilot data, that it was no trouble to enroll families onto the study. I think the ten years that we have tried to create capacity both in complex care and in palliative care, we've really come a long way in terms of treating symptoms.... the capacity to treat and see these kids clinically and also the realization that pain and irritability is a symptom that is worth focusing on has come a long way in that time.” (P1)* |
| *“So I think in some ways it's like all of our clinical nursing experience is that the people who are really sick now, when we see them, they're much sicker than they were before. Whereas back then you could just, you know... You could find these kids quite easily because there were so many more of them. Now there are fewer and they're quite a lot sicker.” (P1)* |
| *“So, obviously not all of those kids [on gabapentin] are kids that are probably eligible for the study, but there might be some. And so those are kids that are being treated for pain or sleep and it's helping their pain or whatever it is. And and doctors are just finding ways to help them, even if it's not been clinically studied.” (P2)* |
| *“…I sometimes wonder, is it because people are getting more aware of these things and are able to assess better and are treated better also?... just advances in technology or assessments...” (P5)* |
| *“…I'm sure medical education, right, it's more focused, a little bit less on I mean, of course, physiology is extremely important. But that psychosocial aspect, like I've noticed a lot of the students coming out of the programs are more focused on that. They are being more empathetic towards pain.” (P3)* |
| *“… maybe it comes back to the medical education. Maybe it's just that everybody's more aware.” (P1)* |
| *“…there has been some good come out of it. Like we are now moving more towards virtual visits and families are very comfortable staying, if you don’t need to come in for an appointment.” (P4)* |
| *“…we're just now so much more comfortable than we were two years ago with like a virtual visit that I don't know if that's playing into it as well…” (P4)* |
| *“I don't think it's only a worry around COVID itself. It's also just... It's really a different... You know, for some of our parents … "Oh, just like loving how we can meet on Zoom. And it really connects me to people." And, you know, it's really a great opportunity for them to have that be normalized and accessible that you meet this way.” (P6)* |
| *“… some of all the online tools that have become so common during the pandemic have shown people that there are other ways to connect meaningfully in a good way, you know, and in a way that we can get used to with with your health care providers and with researchers. So I think there is an expectation that... That research is... Participating in research does not look like coming in and sitting in a clinic.” (P6)* |
| *“Today, I think if people have to sign any paper thing, they're like, ‘What? Is it not something I can just click on line? Like, That's just too much. Like, who's got a printer? Who's going to do that?’” (P6)* |
